# Oligosymptomatic long-term carriers of SARS-CoV-2 display impaired innate resistance but increased high-affinity anti-spike antibodies

**DOI:** 10.1016/j.isci.2023.107219

**Published:** 2023-06-28

**Authors:** Elena Montes-Cobos, Victoria C. Bastos, Clarice Monteiro, João C.R. de Freitas, Heiny D.P. Fernandes, Clarice S. Constancio, Danielle A.S. Rodrigues, Andreza M.D.S. Gama, Vinicius M. Vidal, Leticia S. Alves, Laura Zalcberg-Renault, Guilherme S. de Lira, Victor A. Ota, Carolina Caloba, Luciana Conde, Isabela C. Leitão, Amilcar Tanuri, Orlando D.C. Ferreira, Renata M. Pereira, André M. Vale, Terezinha M. Castiñeiras, Dominique Kaiserlian, Juliana Echevarria-Lima, Marcelo T. Bozza

**Affiliations:** 1 Laboratório de Inflamação e Imunidade, Instituto de Microbiologia Paulo de Góes, Universidade Federal do Rio de Janeiro, Rio de Janeiro, Brazil; 2 Laboratório de Imunologia Básica e Aplicada, Instituto de Microbiologia Paulo de Góes, Universidade Federal do Rio de Janeiro, Rio de Janeiro, Brazil; 3 Laboratório de Biologia de Linfócitos, Instituto de Biofísica Carlos Chagas Filho, Universidade Federal do Rio de Janeiro, Rio de Janeiro, Brazil; 4 Departamento de Doenças Infecciosas e Parasitárias, Faculdade de Medicina, Universidade Federal do Rio de Janeiro, Rio de Janeiro, Brazil; 5 Laboratório de Virologia Molecular, Instituto de Biologia, Universidade Federal do Rio de Janeiro, Rio de Janeiro, Brazil; 6 Laboratório de Imunologia Molecular, Instituto de Microbiologia Paulo de Góes, Universidade Federal do Rio de Janeiro, Rio de Janeiro, Brazil; 7 INSERM U1060, Université Claude Bernard Lyon 1, Centre hospitalier Lyon-Sud, Pierre-Benite, France

**Keywords:** Health sciences, Immunology, Virology

## Abstract

The vast spectrum of clinical features of COVID-19 keeps challenging scientists and clinicians. Low resistance to infection might result in long-term viral persistence, but the underlying mechanisms remain unclear. Here, we studied the immune response of immunocompetent COVID-19 patients with prolonged SARS-CoV-2 infection by immunophenotyping, cytokine and serological analysis. Despite viral loads and symptoms comparable to regular mildly symptomatic patients, long-term carriers displayed weaker systemic IFN-I responses and fewer circulating pDCs and NK cells at disease onset. Type 1 cytokines remained low, while type-3 cytokines were in turn enhanced. Of interest, we observed no defects in antigen-specific cytotoxic T cell responses, and circulating antibodies displayed higher affinity against different variants of SARS-CoV-2 Spike protein in these patients. The identification of distinct immune responses in long-term carriers adds up to our understanding of essential host protective mechanisms to ensure tissue damage control despite prolonged viral infection.

## Highlights

• Immunocompetent oligosymptomatic COVID-19 patients may have persistent infection
• Long-term COVID-19 patients show low antiviral immunity, with fewer NK cells/pDCs
• A systemic type 3 immune profile characterizes persistent SARS-CoV-2 infection
• Long-term carriers develop anti-spike antibodies with enhanced binding capacity

## Graphical abstract

**Figure:**
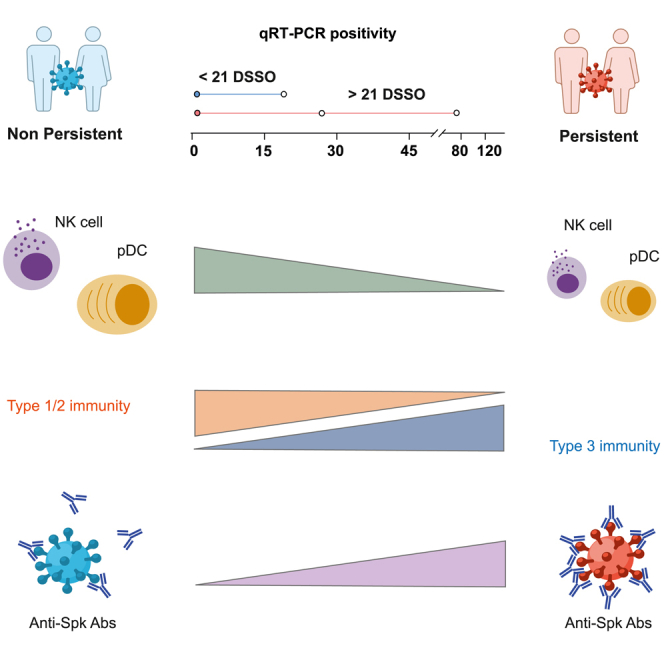

## Introduction

Patients infected with severe acute respiratory syndrome coronavirus 2 (SARS-CoV-2) have clinical presentations ranging from asymptomatic-mildly symptomatic (70–90%) to severe and critical (10–30%).^1^^,^^2^^,^^3^^,^^4^ These different clinical outcomes, including the risk of COVID-19-related death, have been associated with age, gender, and underlying comorbidities, such as obesity and diabetes.^2^^,^^5^^,^^6^ Regardless of pathogen loads, critically ill COVID-19 patients present local and systemic inflammation leading to severe tissue dysfunction, characterized by an increase in inflammatory cytokines, monocytes, and neutrophils, and a marked decrease in lymphocytes compared to patients with mild disease.^7^^,^^8^^,^^9^^,^^10^^,^^11^^,^^12^^,^^13^^,^^14^ Moreover, a significant fraction of patients with life-threatening COVID-19 present defects in type I IFNs (IFN-I) because of inborn mutations and auto-antibodies, pointing to a critical role of IFN-I in the immune response against SARS-CoV-2.^10^^,^^11^^,^^12^ These distinct immune and inflammatory signatures are observed early after COVID-19 diagnosis, correlate with divergent disease trajectories and might have prognostic value.^9^^,^^13^^,^^14^

Alternatively, immunosuppressed individuals, who exemplify the paradigm of low host resistance, display a variety of clinical presentations, from asymptomatic to severe.^15^^,^^16^^,^^17^^,^^18^ Low resistance might impact SARS-CoV-2 clearance in multiple ways, leading to high viral titers in the upper-respiratory tract (URT), dissemination to other tissues, especially the lungs, or long-term virus persistence. Although viral persistence has been more frequently described in immunosuppressed patients, persistent URT infection and long-term virus shedding have been documented in immunocompetent patients with asymptomatic or mild COVID-19 as well.^19^^,^^20^^,^^21^^,^^22^^,^^23^ Most long-term carriers remained SARS-CoV-2 positive by qRT-PCR despite seroconversion, reinforcing the risk of continuous SARS-CoV-2 transmission.^20^^,^^24^^,^^25^ Defects in antigen-specific cytotoxic T cell responses were found in such patients,^26^ but the immune dynamics along the course of infection remain unclear. Thus, the aim of this study is to gain insights into the immune mechanisms associated with prolonged SARS-CoV-2 infection in oligosymptomatic, immunocompetent subjects. Overall, our study reveals alternative immune strategies to cope with SARS-CoV-2 infection, shedding light on the mechanisms of resistance and disease tolerance in COVID-19.

## Results

### Demographic characterization of study cohort

**Figure 1:**
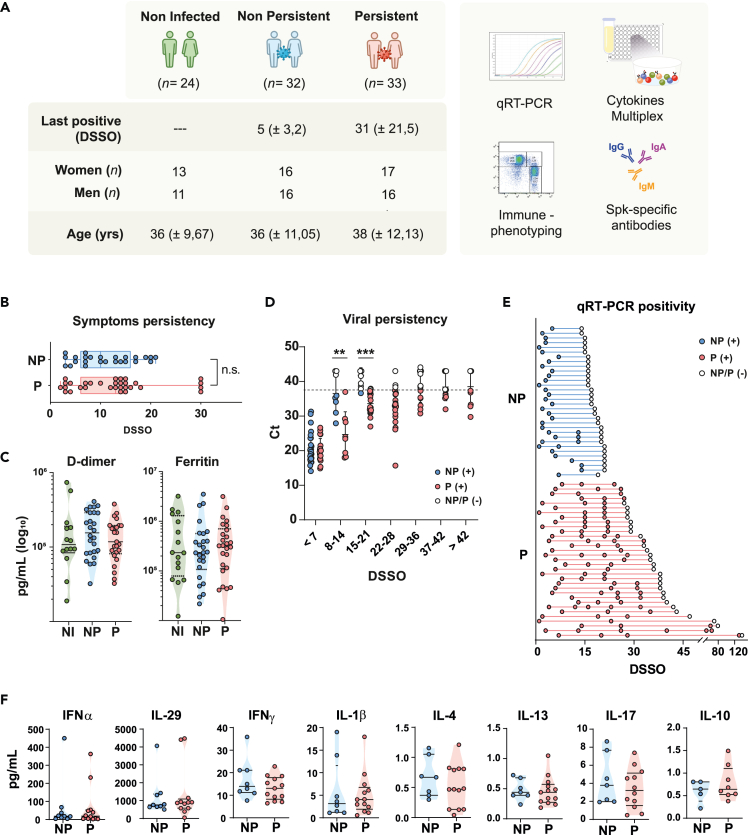
Overview of clinical features, viral loads, and mucosal immunity parameters of COVID-19 patients (A) SARS-CoV-2 positivity by qRT-PCR in nasopharyngeal samples, sex and age of non-infected controls (NI), non-persistent (NP) and persistent (P) COVID-19 patients. (B) Duration of symptoms of NP and P patients, represented as days after symptom onset (DSSO). (C) Quantification of the plasma damage markers D-dimer and Ferritin in NI (n = 14), NP (n = 26), and P (n = 24) at <10 DSSO by multiplex magnetics bead-based immunoassay. (D) Cycle threshold (Ct) values of qRT-PCR for SARS-CoV-2 target genes from nasopharyngeal samples from NP (n = 32) and P (n = 33) patients by DSSO. (E) SARS-CoV-2 positivity of NP (n = 32) and P (n = 33) patients by DSSO. (F) Quantification of IFNα, IL-29, IFNγ, IL-1b, IL-4, IL-13, IL-17, and IL-10 from URT samples in NP (n = 9) and P (n = 13) at <10 DSSO by multiplex immunoassay. Each dot represents a subject. Filled dots represent a positive qRT-PCR of nasopharyngeal samples for SARS-CoV-2, while empty dots represent a negative qRT-PCR. Statistical significance was calculated using Mann-Whitney test, Kruskal-Wallis analysis followed by Dunn post-test, or Holm-Sidak method. ∗∗p ≤ 0.01; and ∗∗∗p ≤ 0.001. DSSO, Days since symptom onset.

Our study cohort is composed of individuals tested for SARS-CoV-2 infection at the diagnostic screening center for COVID-19 of the Federal University of Rio de Janeiro (CTD-UFRJ) from April to December 2020. Weekly follow-up was offered to those subjects who tested positive for the presence of SARS-CoV-2 RNA by quantitative PCR with reverse transcription (RT-qPCR) in nasopharyngeal swab samples, until SARS-CoV-2 RNA was no longer detectable. Initial studies performed in the CTD-UFRJ cohort found a median of SARS-CoV-2 RT-qPCR positivity of three weeks after symptoms onset.^19^ Day 21 since symptom onset (DSSO) was thus used as a putative threshold time point of viral clearance from the URT. From those individuals who volunteered to longitudinal follow-up testing, we selected 33 patients with persistent SARS-CoV-2 infection (P), hereafter referred to as long-term carriers, defined by detectable SARS-CoV-2 RNA (i.e. Ct < 38) at ≥ 21 DSSO. Thirty-two SARS-CoV-2 infected patients with a negative RT-qPCR (i.e., Ct ≥ 38) at ≤ 21 DSSO were also included as COVID-19 control study group, hereafter referred to as non-persistent (NP), and 24 age- and gender-matched asymptomatic, non-infected individuals (confirmed by the absence of spike-specific IgM and IgG in the plasma) were selected as the reference group (NI) (Figure 1A). The proportion of men and women was equally distributed among the study groups. The median age distribution was 36 (±9.67) years of age for the NI group, 36 (±11.05) for those of the NP group and 38 (±12.13) for patients with persistent SARS-CoV-2 infection (P). COVID-19 patients included in this study were either oligosymptomatic, presenting mild symptoms including fever, headache, cough, sneeze, anosmia or myalgia, or asymptomatic at the time of enrollment. Despite prolonged URT infection, long term carriers displayed neither a significantly longer duration of symptoms (Figure 1B) nor augmented disease severity markers in the plasma (Figure 1C). None of the patients of our cohort developed severe COVID-19 requiring hospitalization or oxygen therapy. The most common comorbidities reported by the patients were hypertension (15.63% of NP versus 27.28% of P), hypothyroidism (6.25% NP versus 12.12% of P), and diabetes mellitus (6.25% of NP versus 3.03% of P) (Table 1).

**Table 1:** Demographics of patient cohort

	Non persistent (n = 32)	Persistent (n = 33)	p value
**Characteristics**			

Age, median (years)	36 (±11.05)	38 (±12.13)	0.4606
Sex, female	16/32 (50%)	17/33 (51.52%)	
Last positive, median (DSSO)	5 (±3.2)	31 (±21.5)	<0.0001

**Comorbidities**			

Hypertension	5/32 (15.63%)	9/33 (27.28%)	
Diabetes Mellitus	2/32 (6.25%)	1/33 (3.03%)	
Hypothyroidism	2/32 (6.25%)	4/33 (12.12%)	
Others	4/32 (12.50%)	5/33 (15.16%)	

### SARS-CoV-2 persistency does not depend on viral load or mucosal cytokines

As SARS-CoV-2 enters the organism via the URT, early local immunity at the nasopharyngeal mucosa may be important for fast and efficient viral clearance. Therefore, we first analyzed early viral loads and immune parameters in the nasopharyngeal mucosa of patients that eventually developed prolonged SARS-CoV-2 infection. Viral titers, determined by RT-qPCR, in nasopharyngeal swabs of long-term carriers were similar to those of non-persistent COVID-19 patients during the first days of infection (≤7 DSSO), but started differing after the first week of infection. Most long-term carriers presented detectable viral RNA in the URT for up to 4 weeks, albeit with slightly higher Ct values (Figure 1D). Strikingly, a group of patients still tested positive by RT-qPCR for longer than 8 weeks, with the latest positivity result being at 134 DSSO in one patient (Figure 1E).

Next, we used a multiplex assay to compare a set of 47 immune mediators, including IFNα, IL29, IFNγ, IL-1β, IL-4, IL-13, IL-17A and IL-10, in nasopharyngeal swab samples collected at ≤ 10 DSSO from a group of non-persistent patients (NP, n = 9) and long-term carriers (P, n = 13). The results did not show any differences in early cytokines between both groups (Figures 1F and S1). These results suggest that early alterations of these mucosal cytokines at the entry site of SARS-CoV-2 do not drive viral persistence.

### Type 1 responses shift to type 3 immunity in long-term carriers

**Figure 2:**
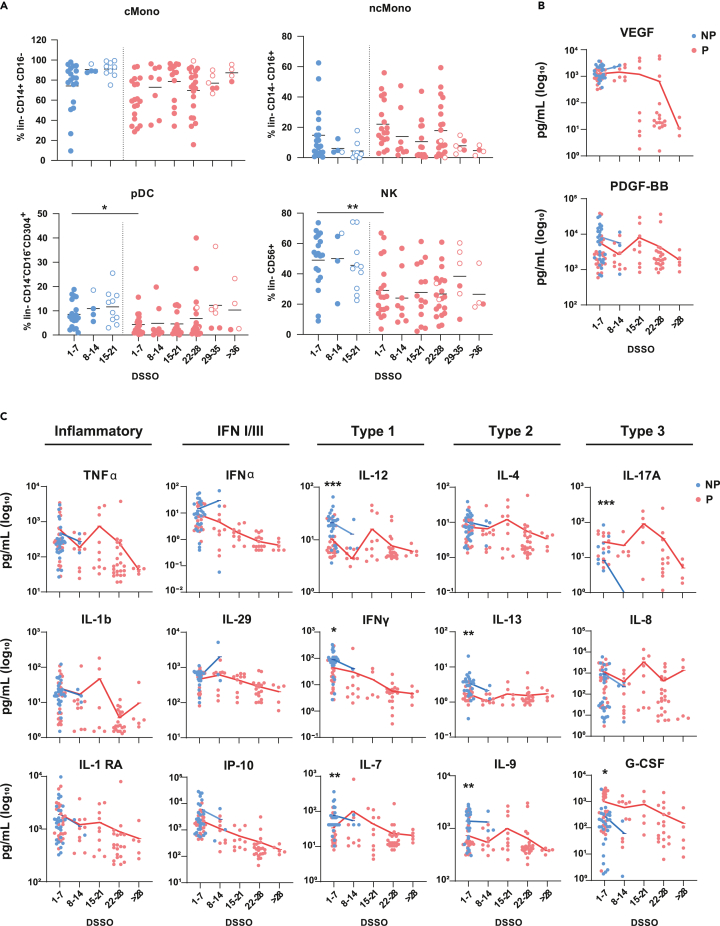
Long-term carriers display type 3-skewed immune responses (A) Longitudinal immunophenotyping of PBMCs from non-persistent (NP, blue, n *=* 28) and persistent (P, red, n *=* 22) patients depicting frequencies of CD14^+^CD16^−^ classical monocytes (cMono) and CD14^−^CD16^+^ non-classical monocytes (ncMono), CD14^−^CD16^−^CD304^+^ plasmacytoid cells (pDC) and CD3^−^CD56^+^ natural killer cells (NK). Filled dots represent individual samples longitudinally collected at different time points until resolution of infection from patients positive by qRT-PCR for SARS-CoV-2, while empty dots represent samples from the same patients at the convalescent phase, coinciding with the first negative qRT-PCR for SARS-CoV-2. (B and C) Weekly longitudinal quantification of plasma growth factors, cytokines and chemokines in non-persistent (NP, blue, n *=* 28) and persistent (P, red, n *=* 22) COVID-19 patients by multiplex immunoassay. Statistical significance was calculated using Kruskal-Wallis analysis followed by Dunn post-test or Mann-Whitney test (onset analysis), and indicated by ∗p ≤ 0.05; ∗∗p ≤ 0.01; and ∗∗∗p ≤ 0.001. DSSO, Days since symptom onset.

The immune system orchestrates distinct resistance mechanisms depending on the nature of the infectious agent, the site of infection and the time after infection onset. Type 1 immunity, mediated by IFNγ, NK cells, T helper 1 (Th1) lymphocytes and cytotoxic T cells, is primarily induced in response to intracellular pathogens, such as viruses. Dysregulated systemic inflammatory and antiviral responses have been pointed out as potential drivers of distinct clinical progression of COVID-19.^9^^,^^10^^,^^13^ Therefore, to gain insights into specific immune mechanisms leading to prolonged SARS-CoV-2 infection, we compared innate immune cells by flow cytometry longitudinally in non-persistent COVID-19 patients and long-term carriers, starting early after disease onset (≤10 DSSO). Frequencies of circulating monocyte populations, including classical (lin^−^CD14^+^CD16^−^), intermediate (lin^−^CD14^+^CD16^+^) and non-classical (lin^−^CD14^−^CD16^+^) monocytes, were similar in both groups across the infection, and so were the frequencies of dendritic cells (DCs) (Figures 2A, S2, and S3A). However, we found a significant reduction in the percentage of blood-circulating pDCs (lin^−^CD14^−^CD304^+^) in long-term carriers. The percentage of circulating NK cells (lin^−^CD56^+^) was also reduced in patients with long-term infection compared to non-persistent ones and remained low over the infection period and on the convalescent phase. Altogether, these results suggest a deficient activation of type 1 immunity in long-term SARS-CoV-2 infection.

Next, we titrated plasma levels of tissue growth factors like PDGF-BB, basic FGF and VEGF, which are involved in tissue damage and repair and have been previously connected to disease tolerance.^27^ We did not observe any increase in any of these factors in long-term carriers, indicating that systemic tissue repair mechanisms were not preferentially induced in these patients (Figures 2B and S4A). While VEGF concentrations acutely dropped at the second week after symptom onset, some patients showed a transient increase in PDGF-BB and basic FGF around this time point and stabilized later on. The analysis of inflammatory mediators previously found to correlate with COVID-19 symptoms, such as TNFα, IL-1β or IL-1 RA,^7^^,^^8^^,^^9^ showed that long-term carriers display inflammatory responses comparable to non-persistent patients at the onset of disease, which mildly increased at the third week of infection in some of the patients (Figure 2C). These results suggest that the absence of sustained symptoms or tissue damage in prolonged SARS-CoV-2 infection is probably because of the lack of strong inflammation, and not related to an enhancement of disease tolerance through increased tissue repair.

Furthermore, we aimed to analyze a set of cytokines and chemokines related to type 1, 2 and 3 responses early after disease onset and longitudinally. Since pDCs are the first and major IFN-I producers during viral infections^28^^,^^29^ and were less represented in long-term carriers, it was not unexpected that plasma concentrations of IFNα and the IFN-induced chemokine IP-10 (CXCL10) did not increase in this group of patients (Figure 2C). Strikingly, other type 1 cytokines, such as IL-12, IFNγ and IL-7, were significantly lower in long-term carriers compared with regular COVID-19 patients at disease onset (<7 DSSO). Type 2 cytokines were similarly reduced in long-term carriers, whereas type 3 cytokines related to neutrophil production and recruitment, such as IL-17A and G-CSF, displayed a significant enhancement. Altogether, these results indicate that patients with persistent SARS-CoV-2 infection present an altered immune profile skewed toward type 3 responses.

To visualize the interplay between the analyzed cytokines and viral loads, we depicted cytokine concentrations along with Ct values (represented as Ct-^1^) for the eight individual patients with more than three longitudinal samples (Figure S5A). Instead of a unique immune profile, we found distinct cytokine and Ct dynamics, which suggests that each long-term carrier copes with viral persistence in a different manner.

### Persistent SARS-CoV-2 infection does not affect effector or memory T cell responses

**Figure 3:**
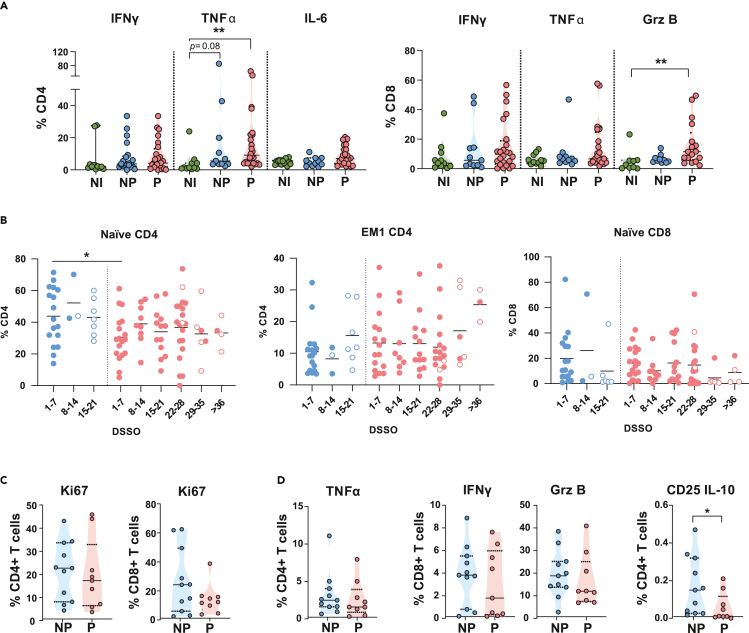
T cell effector and memory responses of persistent patients (A) Frequencies of CD4^+^ T lymphocytes producing IFNγ, TNFα and IL-6 and CD8^+^ T lymphocytes producing IFNγ, TNFα and Granzyme B (Grz B) after polyclonal *in vitro* stimulation of PBMCs from NI, NP and P (≤10 DSSO) with anti-CD3/CD28 beads of PBMCs from (n = 12), NP (n = 11) and P (n = 23). (B) Longitudinal immunophenotyping of PBMC from NP and P depicting CD4^+^CD27^+^CD45RA^+^CCR7^+^ naive T cells (naive CD4), CD4^+^CD27^+^CD45RA^−^CCR7^-^ effector memory T cells (EM1) and CD8^+^CD27^+^CD45RA + ^−^CCR7^+^ naive T cells (naive CD8). Filled dots represent individual samples longitudinally collected until resolution of infection from NP and P patients positive by qRT-PCR for SARS-CoV-2. Empty dots represent samples from convalescent patients coinciding with the first negative qRT-PCR for SARS-CoV-2. (C and D) Immunophenotyping of SARS-CoV-2 reactive CD4 and CD8 T cells after *in vitro* stimulation of PBMC from NP (n = 11) and P (n = 9) after viral clearance with peptides spanning the Spike protein of the alpha variant of SARS-CoV-2 in the presence of IL-2. Percentage of (C) CD4^+^ and CD8^+^ cells expressing Ki67, (D) CD4^+^ cells expressing TNFα, CD8^+^ cells expressing IFNγ and Granzyme B (Grz B), and CD4^+^CD25^+^ cells expressing IL-10. Statistical significance was calculated using Kruskal-Wallis analysis followed by Dunn post-test, and indicated by ∗p ≤ 0.05; ∗∗p ≤ 0.01. DSSO, Days since symptom onset.

Next, we studied the functionality of the T cell compartment in long-term carriers. We analyzed the cytokine production capacity of circulating CD4^+^ and CD8^+^ T cells from the PBMCs of non-infected individuals, non-persistent patients and persistently infected COVID-19 patients at early time points of infection (≤10 DSSO). To that aim, we performed *in vitro* polyclonal stimulation of PBMCs with anti-CD3/anti-CD28 magnetic beads, and analyzed intracellular cytokine production by flow cytometry. Although the production of TNFα by CD4^+^ T cells and Granzyme B by CD8^+^ T cells was enhanced in long-term SARS-CoV-2 carriers compared with the healthy control group, no significant differences in cytokine production were found between non-persistent patients and long-term carriers (Figure 3A). In addition, we analyzed whether the frequencies of circulating memory T cell subpopulations were altered during the course of disease, but we could not find differences between both groups of patients, neither during infection nor at the convalescent phase (Figures 3B and S3). Only naive CD4^+^ T cells appeared to be reduced during the first week of infection in long-term carriers. Furthermore, to elucidate whether long-term infection had or not an effect on the development of antigen-specific T cell responses, we took PBMCs at a DSSO corresponding to the first negative swab PCR result, corresponding to the convalescent phase, and stimulated them with a pool of peptides spanning the sequence of the S protein of the circulating variant of SARS-CoV-2 in Rio de Janeiro at the time of collection. CD4^+^ and CD8^+^ T cells from non-persistent and persistently-infected patients proliferated similarly in response to antigen-specific stimulation, according to the amount of Ki67, detected by flow cytometry (Figure 3C). Production of effector cytokines, such as TNFα, IFNγ, and granzyme B, was also comparable between both groups. However, the frequency of CD25^+^ IL-10 producing cells among the total pool of CD4^+^ cells, representative of Treg populations, was significantly lower in the PBMCs from long-term carriers, suggesting weaker T cell-mediated regulatory responses in this group of patients. Altogether, these results indicate that persistent infection does not interfere with the development of antigen-specific T cell-mediated immune memory.

### Patients developing persistent infection display an early distinct immune signature

**Figure 4:**
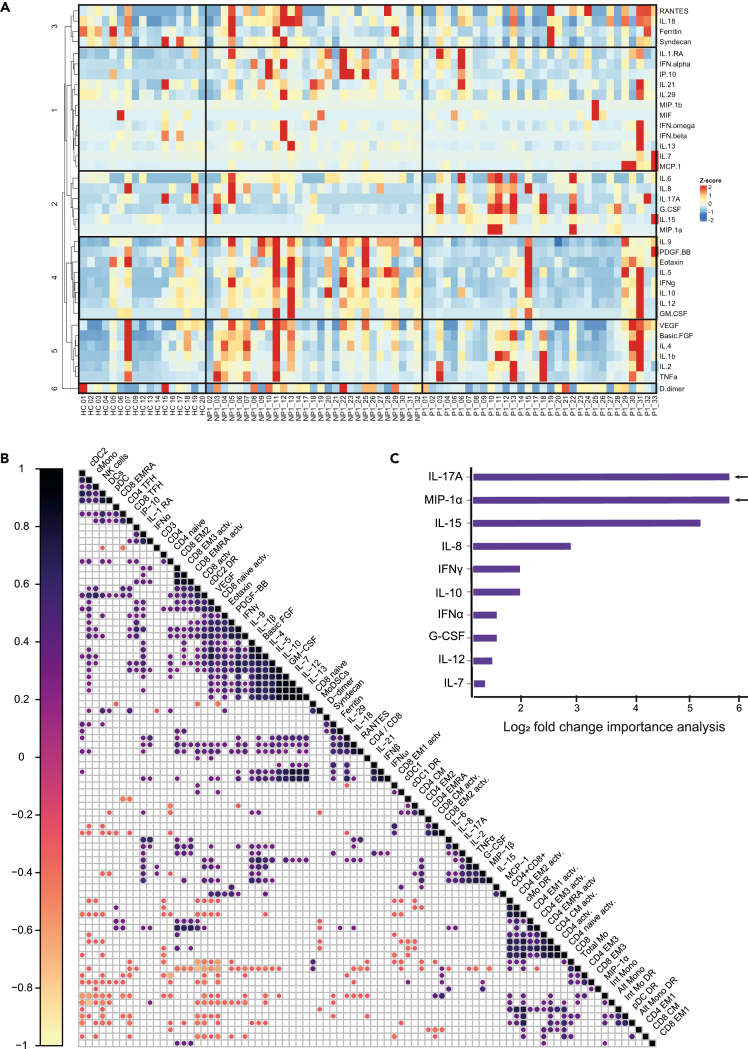
Early immune signature of persistent patients (A) Heatmap of cytokine concentration in serum from NI, NP and P at <10 DSSO measured by multiplex assay. K-means clustering was used to determine cytokine clusters 1–6. (B) Correlation matrix across PBMC immunophenotyping and cytokines concentrations from NI, NP and P at <10 DSSO. Only significant correlations (p < 0.05) are represented as dots. Pearson’s correlation coefficients from comparisons of cytokine measurements within the same patients are visualized by color intensity. (C) Fold change importance analysis between NP and P, calculated using the gtools package in R. IL-17A and MIP-1α have median equals zero in NP, resulting in an infinite fold change, which is represented by the arrow.

Unsupervised cluster analysis of plasma cytokines at ≤ 10 DSSO revealed at least two groups of immune mediators that were differentially regulated in non-persistent patients compared to those with long-term infection (Figure 4A). Cluster 2, composed of IL-6, IL-8, IL-17A, G-CSF, IL-15 and MIP-1α, was overrepresented in most long-term carriers, whereas the cytokines from cluster 4, containing IL-9, IL-10, IFNγ and IL-12, were reduced. In addition, we combined plasma cytokine data with immunophenotyping data from the same time points (Figures 4B, S6A, and S6B). The correlation matrix of soluble proteins and immune cell subtypes revealed a positive correlation of effector CD8^+^ T cells with Th1 and Th2 cytokines which, in turn, inversely correlated with the activation of alternative monocytes and pDCs (Figure 4B). Furthermore, unsupervised cluster analysis on cytokine data by t-distributed Stochastic Neighbor Embedding (t-SNE) identified four different clusters of patients (Figure S6B). All non-infected controls gathered in one cluster, whereas long-term carriers were distributed in three different groups along with non-persistent patients, irrespective of age, gender or comorbidities. Finally, fold change importance analysis performed on NP versus P patients highlighted IL-17A, MIP1α, IL-15, IL-8 and IFNγ as the top five cytokines characterizing SARS-CoV-2 persistency (Figure 4C). Altogether, bioinformatic analysis strongly suggests that a combination of early blood markers correlates with prolonged SARS-CoV-2 infection.

### SARS-CoV-2 persistency favors the generation of high affinity spike-specific antibodies

**Figure 5:**
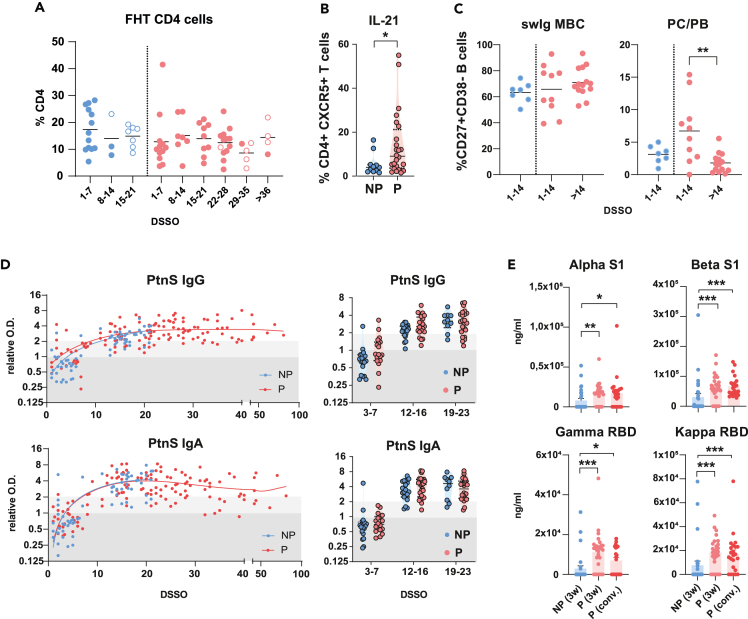
Humoral responses are altered in long-term carriers of SARS-CoV-2 (A) Longitudinal immunophenotyping of PBMC from NP and P depicting CD3^+^CD4^+^CXCR5^+^ follicular T (T_FH_) cells. Filled dots represent individual samples longitudinally collected until resolution of infection from NP and P patients positive by qRT-PCR for SARS-CoV-2. Empty dots represent samples from convalescent patients coinciding with the first negative qRT-PCR for SARS-CoV-2. (B) Percentage of CD4^+^CXCR5^+^ T cells expressing IL-21 after polyclonal *in vitro* stimulation of PBMC from NP (n = 11) and P (n = 24) at <14 DSSO with anti CD3/CD28 beads. (C) Longitudinal immunophenotyping of PBMC from NP (n = 7) and P (n = 10) depicting CD27^+^CD38^−^ switched immunoglobulin (swIg) memory B cells (MBC) and plasmablast/plasma cells (PB/PC). (D) Longitudinal assessment of Spike-specific IgG and IgA antibodies in plasma from P and NP by ELISA assay. (E) Titers of high-affinity antibodies against Alpha S1, Beta S1, Gamma RBD, and Kappa RBD variants of SARS-CoV-2 in plasma from NP and P around 21 DSSO, and P by the time of viral clearance, by multiplex neutralization assay. Statistical significance was calculated using Mann-Whitney test or Kruskal-Wallis analysis followed by Dunn post-test and indicated by ∗p ≤ 0.05; ∗∗p ≤ 0.01; and ∗∗∗p ≤ 0.001. DSSO, Days since symptom onset.

Adequate innate immune activation is essential for the development of adaptive immune responses, but our results showed that long-term carriers presented an altered innate immune profile. As B cell memory and SARS-CoV-2-specific antibody responses might also correlate with distinct disease trajectories, we additionally studied the development of antigen-specific humoral responses in long-term carriers. We started analyzing follicular helper T cells (T_FH_), since they contribute to humoral immunity by delivering to B cells the necessary signals to enter the germinal center, going through class-switch recombination and affinity maturation. Although we did not find changes in the frequencies of circulating CD4^+^ or CD8^+^ T_FH_ cells (Figure 5A), polyclonal stimulation of PBMCs collected at ≤ 14 DSSO revealed that this T cell population produced higher amounts of IL-21 in long-term carriers, being this cytokine essential for B cell help (Figure 5B). Furthermore, we compared the composition of the B cell compartment and antibody development in patients with regular or delayed resolution of infection. We did not observe significantly different frequencies of circulating mature, class-switched B cells, although the proportion of circulating plasmablasts/plasma cells dropped in long-term carriers after 14 DSSO (Figure 5C). Serological analysis did not identify significant alterations in the production of nucleocapsid (N) protein-specific IgG (Figure S7A) or spike (S) protein-specific IgM, IgG and IgA antibodies over time (Figures 5D and S7A). However, plasma of long-term carriers displayed higher titers of circulating high-affinity Spike-specific antibodies against all analyzed SARS-CoV-2 variants already three weeks after symptoms onset, and at least until the patients reached the convalescent phase (Figures 5E and S7B). These data suggest that generation of systemic N protein-specific or S protein-specific humoral responses do not seem to be sufficient for viral clearance during primary SARS-CoV-2 infection, although it might confer better protection against subsequent re-infections.

## Discussion

Our cohort comprises a group of oligosymptomatic patients with persistent infection. Here we show that low resistance in these patients is likely because of impaired innate antiviral immunity, as no major defects in adaptive immunity were found. Furthermore, we observed divergent cytokine profiles in the nasopharyngeal mucosa and in the plasma, in line with other studies recently published.^30^ In contrast with systemic cytokines, we did not find changes in nasopharyngeal immune mediators. However, although relevant mucosal cytokines, such as IL-17A and IL-10, were not altered in the nasopharynx, we cannot exclude the contribution of cytokines with only transient induction or other mucosal mediators not studied here.

Long-term carriers displayed decreased frequencies of circulating pDCs and NK cells shortly after symptom onset and in later time points. The pDCs are considered essential for the control of viral replication through the rapid release of IFN-I.^28^^,^^29^^,^^31^ Early IFN-I triggers different mechanisms targeting virally infected cells, namely the expression of antiviral proteins, the activation of NK cells and the initiation of Th1 responses. In our study, some of these elements were underrepresented in those COVID-19 patients who later on presented persistent infection, indicating that low antiviral innate immune responses may be a possible reason for viral persistency.

Major differences in systemic cytokines, chemokines and growth factors were found in patients with prolonged course of infection from early on. Long-term carriers displayed an immunological shift characterized by a low systemic Th1/2 signature, increasing in turn IL-17A, IL-8 and other neutrophil-recruiting chemokines. In line with our data, mouse models of chronic infection by TMEV also link Th17 cells to viral persistence.^32^ The origin of type 3 cytokines in persistent patients is unclear at this point. Mucosal sites, including the gut, are a major source of IL-17A and highly dependent on the microbiota.^33^ Considering that gut mucosal tissues are sites of active SARS-CoV-2 replication,^34^ it is possible that the observed increase in type 3 cytokines might be influenced by the microbiome, and by viral persistence at these sites.

Impaired IFN-I responses and an enhanced type 3 signature are also traits that had been previously associated with severe COVID-19.^9^^,^^10^^,^^11^ Conversely, inflammatory and Th1/2 responses are elevated in patients with severe symptoms but low in oligosymptomatic long-term carriers, which suggests their involvement in the pathogenesis of COVID-19.^7^^,^^8^^,^^9^^,^^13^ All in all, our data suggest that early systemic immunological patterns may indicate future persistency of SARS-CoV-2 infection in immunocompetent patients. Further studies to better characterize the correlation of these immunological patterns with infectious disease outcome are warranted.

The engagement of disease tolerance mechanisms, like enhanced tissue repair, metabolic adaptations or immune regulation, may also lead to distinct disease trajectories.^35^^,^^36^^,^^37^ Our data could not identify enhanced systemic tissue repair or regulatory responses during the course of prolonged SARS-CoV-2 infection. Actually, the immunoregulatory cytokine IL-10, which is commonly linked to immunological tolerance, but also a well-accepted disease marker in COVID-19,^38^^,^^39^ was particularly low in long-term carriers. In order to understand the physiological meaning of this effect and the involvement of additional disease tolerance mechanisms, further analysis should be conducted.

In our study, long-term SARS-CoV-2 carriers had higher titers of spike-specific antibodies already three weeks after symptom onset. First, this suggests that systemic high affinity antibodies against Spike/RBD may not be sufficient for viral clearance from the URT during primary infection. Type I IFNs might contribute to this effect, since they have been shown to regulate B cell responses and antibody production in the context of viral infection.^40^^,^^41^ Furthermore, patients with severe COVID-19, who may remain infected for extended time periods and present dysregulated IFN-I and type 3 responses, have been shown to produce higher titers of neutralizing antibodies.^42^ Together with our data, this suggests that dampened IFN-I responses and/or strong type 3 immunity in persistent SARS-CoV-2 infection might confer advantages against re-infection. On the other hand, these antibodies could also contribute to viral persistence via antibody-dependent enhancement or modulate COVID-19 immunopathology via Fc-receptors. Hence, analysis of the functionality of the antibodies elicited during long-term infection, and longer follow-up of the adaptive cellular and humoral immunity in these patients could be valuable for COVID-19 treatment and vaccine design and development.

Collectively, our study provides a thorough analysis of the immune dynamics during viral persistency in COVID-19 patients. Over the past three years, important progress has been made in controlling the pandemic, but the vaccines available, though successful in limiting disease, are not sterilizing and do not completely prevent transmission of new SARS-CoV-2 variants.^43^ In COVID-19 as well as in other viral infections, oligosymptomatic and asymptomatic carriers represent the main vector of viral transmission. Therefore, long-term immunocompetent infected individuals are potential long-term spreaders and may, in addition, facilitate intra-host evolution of SARS-CoV-2 and other viruses.^15^^,^^19^^,^^20^ This reinforces the need for a better understanding of the immune mechanisms of viral control in these patients. Shedding light on this issue, our study identifies a set of early plasma markers associated with prolonged infection and reveals alternative immunological strategies to deal with viral infection without overt tissue damage and pathology.

### Limitations of the study

Given the out-care characteristic of our cohort and the techniques available, our investigation of viral RNA was restricted to nasopharyngeal samples. Assessment of viral titers in various tissues would allow for a better understanding of whether long-term carriers display systemic low resistance, or if this phenomenon is restricted to the nasopharyngeal mucosal site. Although we did not find increased damage and tissue repair markers in the blood or in the nasal mucosa, we cannot exclude that other SARS-CoV-2 target tissues, such as the lungs or the gut, have altered disease tolerance or support viral replication. To study disease tolerance and viral presence in such locations, animal models for SARS-CoV-2 would be helpful. Furthermore, our samples are limited and were collected in a specific timing and geographical context of the pandemic. Nonetheless, to our knowledge, the present study constitutes the characterization of immune parameters in the larger number of immunocompetent oligosymptomatic patients with persistent infection of SARS-CoV-2 so far. Still, sample size is critical when studying such a heterogeneous disease as COVID-19, and, therefore, validation of our data in larger, independent cohorts would be informative.

## STAR★Methods

### Key resources table

**Table:** 

REAGENT or RESOURCE	SOURCE	IDENTIFIER
**Antibodies**		

IgD-PE-Cy7 Clone: IA6-2	Biolegend	348210
CD15-BV421 Clone: W6D3	Biolegend	323040
CD56-PE-Dazzle™ 594 Clone: HCD56	Biolegend	318348
CD14-PECy5 Clone:M5E2	Biolegend	301864
CD16-BV786 Clone:3G8	Biolegend	302046
HLA-DR-BB515/PECy5 Clone: G46-6	BD Bioscience	555813; 564516
CD11b-APC-Cy7 Clone: ICRF44	Biolegend	301352
CD304-PE Clone: 12C2	Biolegend	354504
CD11c-BV711 Clone: 3.9	Biolegend	301630
CD1c-AF647 Clone: L161	Biolegend	331510
CD141-BV650 Clone: 1A4	BD Bioscience	740604
CD3-BV510/BV605 Clone: UCHT1	Biolegend	300448; 300468
CD4-BV785 Clone:SK3	Biolegend	344642
CD19-BV421/BV510 Clone HIB19	Biolegend	302233; 302242
CD8-BV711/APC-Fire750 Clone: SK1	Biolegend	344746
CD27-FITC Clone: O323	Biolegend	302806
CCR7-BV421 Clone: G043H7	Biolegend	353208
CD45RA-PE-Cy7/PE-Cy5 Clone: HI100	Biolegend	304126; 304110
CD25-AF700 Clone: BC96	Biolegend	302622
CD38-BV711 Clone: HIT2	Biolegend	303528
CXCR5-PE-Dazzle™ 594 Clone: J252D4	Biolegend	356928
Ki67-AF647 Clone: Ki-67	Biolegend	350510
IL-10-BV421 Clone: JES3-9D7	Biolegend	501422
Granzyme B-FITC Clone: GB11	BD Bioscience	560211
IFNγ-APC-Fire750 Clone: 4S.B3	Biolegend	502548
TNFα-BV650 Clone: MAb11	Biolegend	502938
IL-6-PE-Cy7 Clone: MQ2-13A5	Biolegend	501120
IL-21-PerCP-Cy5.5 Clone: 3A3-N2	Biolegend	513012

**Chemicals, peptides, and recombinant proteins**		

anti-CD3/anti-CD28 DynabeadsTM Human T-activator	Gibco™	11131D
PepMix™ SARS-CoV-2 (Spike Glycoprotein)	JPT	PM-WCPV-S-1
Recombinant Human IL-2 (carrier-free)	Biolegend.	589104
Brefeldin A solution	Biolegend	420601
LIVE/DEAD Fixable Aqua Dead Cell staining	Invitrogen™	L34957

**Critical commercial assays**		

Maxwell 16 viral total nucleic acid purification kit	Promega	AS1150
SARS-CoV-2 (2019-nCoV) CDC qPCR probe assay	Integrated DNA Technologies	10006713
Bio-Plex Pro™ Human Cytokines 27-Plex Assay	BIO-RAD	M500KCAF0Y
Human ProcartaPlex™ kit	Invitrogen™	PPX-11
Bio-Plex Pro Human SARS-CoV-2 Neutralization Antibody Assay	BIO-RAD	17007632

**Deposited data**		

Multiplex plasma cytokine and PBMC flow cytometry analysis long-term COVID-19 patients	Mendeley Data	https://doi.org/10.17632/gxmnms9whf.1

**Software and algorithms**		

FlowJo™ v10	BD Bioscience	
xPONENT®	Luminex	
SoftMax	Molecular Devices	
GraphPad Prism 8	Dotmatics	

### Resource availability

#### Lead contact

Further information and requests for resources and reagents should be directed to and will be fulfilled by the lead contact, Marcelo Torres Bozza (mbozza@micro.ufrj.br).

#### Materials availability

This study did not generate new unique reagents.

### Experimental model and study participants details

All patients included in the present study sought testing at the Diagnostic Screening Center for COVID-19 at the Federal University of Rio de Janeiro (CTD-UFRJ) and declared written informed consent. From April to December 2020, we enrolled two thousand seven hundred and fifty-nine patients who were tested for SARS-CoV-2 infection at the Diagnostic Screening Center for COVID-19 of the Federal University of Rio de Janeiro (CTD-UFRJ). Among them, 1,133 individuals (41.07%) tested positive for the presence of SARS-CoV-2 RNA by quantitative PCR with reverse transcription (RT-qPCR) on nasopharyngeal swab samples. Those individuals were offered weekly follow-up testing until SARS-CoV-2 RNA was no longer detected. Blood from those patients was collected in heparinized tubes for plasma and PBMC storage and further analysis. Symptoms, use of medication, comorbidities and demographic information were assessed by oral questionnaire performed by trained personnel. Based on blood sample availability, 33 patients were selected from those with persistent SARS-CoV-2 infection, defined as positive SARS-CoV-2 RT-qPCR in upper respiratory tract (URT) samples after 21 days after symptom onset. As such, 32 patients were selected from those who did not display persistent viral RNA, defined as a negative SARS-CoV-2 RT-qPCR in URT samples up to 21 days after symptom onset. The criterion was guided by the median of positivity duration in the overall cohort, which was around three weeks (Voloch et al, 2021). Twenty-five non-infected volunteers were included as controls, defined as a negative SARS-CoV-2 qRT-PCR, no history of a positive SARS-CoV-2 qRT-PCR and no seroconversion for SARS-CoV-2 epitopes. Since age and gender of the study subjects may influence the obtained results, all groups were matched by median age and male and female individuals were equally distributed (Table 1). All procedures and experiments were approved by the correspondent Ethic Committee Board (CAAE: 30161620.0.1001.5257; 4.245.490).

### Method details

#### PBMC and plasma isolation

PBMCs were isolated from blood collected in lithium heparin tubes by Ficoll–Hypaque density gradient centrifugation. Briefly, blood was layered on a density gradient (Hystopaque® 1077, Sigma-Aldrich), and PBMCs were separated by centrifuging at 400 rcf for 30 min. PBMCs were washed four times in phosphate-buffered saline (PBS), submitted to ACK for 5 min to lysate red blood cells, and frozen in liquid nitrogen in 90% fetal bovine serum (FBS) with 10% DMSO (Sigma-Aldrich) until used for flow cytometry and stimulation assays. Plasma samples were collected in 10-ml tubes in lithium heparin tubes, centrifuged at 400 rcf for 10 min, aliquoted, and stored at −20°C for further experiments.

#### RNA isolation and RT-qPCR

Total viral RNA was extracted from swab samples using the Maxwell 16 viral total nucleic purification kit System (Promega) according to manufacturer’s instructions. Viral RNA was detected using the SARS-CoV-2 (2019-nCoV) CDC qPCR probe assay (Integrated DNA Technologies, IA, USA) targeting the SARS-CoV-2 N1 and N2 genes and the human RNase P (RNaseP) gene. All reactions were paired and performed in a 7500 thermal cycler (Applied Biosystems, CA, USA). A SARS-CoV-2 qRT-PCR result was considered positive if both targets (N1 and N2) were amplified with a cycle threshold (CT) of ≤40, inconclusive if only one target was amplified with a CT of #40, and negative if both targets were not amplified or amplified with a CT >40.

#### Cell culture

PBMCs were thawed in RPMI 1640 medium (Lonza) supplemented with 10% FBS (Gibco™) and penicillin (100 IU/ml; Gibco™)/streptomycin (100 μg/ml; Gibco™). For T cell stimulation, 0.5×10^6^ PBMCs were cultured in 500μl of medium in a 24-well flat-bottomed microplate and stimulated for 4 days with anti-CD3/anti-CD28 Dynabeads™ Human T-activator (10 μL/mL, Gibco™). For detection of SARS-CoV-2–specific CD4^+^ and CD8^+^ T cells, 0.5×10^6^ PBMC cells were stimulated in a 96-well U-bottom plate for 7 days in 200 μl of medium containing 1 μg/ml of PepMix™ SARS-CoV-2 (Spike Glycoprotein) (JPT Peptide Technologies) in the presence of IL-2r (20UI, Biolegend). As a control, some cells were maintained with IL-2r alone. After 6 days, the cells were re-stimulated with 10μg/ml of the aforementioned peptide pools overnight. In order to optimize the detection of intracellular cytokines, Brefeldin A (10 μg/mL; Biolegend) was added in the last 4 h (for Dynabeads™) or 12h (for SARS-CoV-2 peptide pools) of the cell cultures. Cultures were maintained in a humidified incubator with 5% CO2 at 37°C. After stimulation, cells were stained for proliferation and/or phenotypic lymphocyte markers by Flow Cytometry.

#### Flow cytometry

Staining for flow cytometry analysis was performed using fluorescently-labeled specific anti-human antibodies. Unless otherwise stated, antibodies were purchased from Biolegend: IgD-PE-Cy7, CD15-BV421, CD56-PE- Dazzle™594, CD14-PECy5, CD16-BV786, HLA-DR-BB515/PECy5 (BD Bioscience), CD11b-APC-Cy7, CD304-PE, CD11c-BV711, CD1c-AF647, CD141-BV650, CD3-BV510/BV605, CD4-BV785, CD19-BV421/BV510, CD8-BV711/APC-Fire750, CD27-FITC/PE, CCR7-BV421, CD45RA-PE-Cy7/PE-Cy5, CD25-AF700, CD38-BV711/PE-Cy5, CXCR5-PE- Dazzle™594, Ki67-AF647, IL-10-BV421, Granzyme B-FITC (BD Bioscience), IFNγ-APC-Fire750, TNFα-BV650, IL-6-PE/PE-Cy7, and IL-21-PerCP-Cy5.5.

Briefly, 5x10^5^ cells were incubated with LIVE/DEAD Fixable Aqua Dead Cell staining (1:1000, BV510; Invitrogen) in PBS for 30 min, and washed with PBS containing 3% FBS and 0.01% sodium azide. Next, cells were incubated with the fluorescently-labelled antibodies for 30 min at room temperature in the dark. When intracellular staining was required, cells were permeabilized with the Cytofix/Cytoperm solution (BD Pharmigen) at 4°C for 20 min and subsequently incubated for 30 min at 4°C with the appropriate antibodies. Events were acquired on LSR FORTESSA X-20 (BD Biosciences). Target cells were gated based on forward and side scatter properties, singlets and living cells were analyzed by using FlowJo™ v10 Software (Figures S2A–S2C). FMO controls and single-stained samples were used to periodically check the settings and gates on the flow cytometer.

#### Quantification of inflammatory, anti-inflammatory, and antiviral mediators

Cytokines and immune mediators were quantified with a multiplex magnetic bead-based immunoassay according to the manufacturer’s instructions, using the Bio-Plex Pro™ Human Cytokines 27-Plex Assay (BIO-RAD) and the Human ProcartaPlexTM kit (Invitrogen™, ThermoFisher Scientific), including the following analytes: basic FGF, Eotaxin, G-CSF, GM-CSF, IFN-γ, IL-1β, IL-1RA, IL-2, IL-4, IL-5, IL-7, IL-8, IL-9, IL-10, IL-12 (p70), IL-13, IL-15, IL-17A, IP-10/CXCL10, MCP-1/CCL2, MIP-1α/CCL3, MIP-1β/CCL4, PDGF-BB, TNF-α, and VEGF, IFNα, IFNβ, IFNΩ, IL-29, IL-18, IL-21, syndecan, ferritin. Data were acquired in a Luminex MAGIPIX® System (serial number MAGPX11266003) using the xPONENT software. Additionally, IL-6, and RANTES/CCL5 concentrations were determined in plasma samples by enzyme-linked immunosorbent assay (Human IL-6 and RANTES/CCL5 TMB ELISA, Peprotech), according to the manufacturer’s instructions. Absorbance was measured in a SpectraMax® microplate reader (Molecular Device).

#### SARS-CoV-2 specific immunoglobulins and neutralizing antibodies

Anti-SARS-CoV-2 spike protein IgM, IgA, and IgG antibodies were quantified by enzyme-linked immunosorbent assay, following the *S-UFRJ test* protocol, as previously described.^44^ Briefly, serial dilutions of plasma samples (starting 1:40, in PBS 1% BSA) were incubated in high-binding ELISA plates previously coated with 50 μL of SARS-CoV-2 spike protein (4 μg/mL) overnight. Antibodies were detected with goat anti-human IgG, IgA, and IgM (Fc)-horseradish peroxidase antibodies (Southern Biotech) and developed with TMB (Scienco). Optical density (OD) was measured in a SpectraMax® microplate reader (Molecular Device, USA). Plasma titers of neutralizing antibodies were quantified with a multiplex magnetic bead-based immunoassay, using the Bio-Plex Pro Human SARS-CoV-2 Neutralization Antibody Assay (BIO-RAD) according to the manufacturer’s instructions.

### Quantification and statistical analysis

Description of the number of experimental subjects per group (*n*) and statistical analysis performed for each set of data can be found in the figure legends. One-dimensional probability distributions of samples were analyzed by Kolmogorov–Smirnov test. Statistical analysis was performed by one-way variance analysis followed by Bonferroni post-test for samples with normal distribution, or, alternatively Kruskal-Wallis analysis followed by Dunn post-test. For comparison of two groups on longitudinal Ct values, multiple Student’s t test analysis was performed, using the Holm-Sidak method. Statistical significance was calculated and defined as ∗ *p* ≤ 0.05; ∗∗ *p* ≤ 0.01; and ∗∗∗ *p*≤ 0.001. Correlations were analyzed by Spearman’s rank correlation coefficient. The statistical analysis was performed using Prism 8.0 software (GraphPad Software, San Diego, CA).

## Data Availability

• Luminex and flow cytometry data reported in this paper is publicly available on Mendeley Data repository (https://data.mendeley.com/datasets/gxmnms9whf) as of the date of publication. • Original code and additional Supplemental Items are available from Mendeley Data at https://data.mendeley.com/datasets/gxmnms9whf. • Any additional information required to reanalyze the data reported in this paper is available from the lead contact upon request.

## Supplemental information

Document S1. Figures S1–S7

Data S1. Original code used in this study, related to Figure 4
