# Supplementary material for: Oligosymptomatic long-term carriers of SARS-CoV-2 display impaired innate resistance but increased high-affinity anti-spike antibodies
Source: iScience. 2023 Jun 28;26(7):107219. doi: 10.1016/j.isci.2023.107219 (PMC10300054; doi:10.1016/j.isci.2023.107219)
Supplement: Document S1. Figures S1–S7 [file mmc1.pdf]

iScience, Volume *26*

Supplemental information

Oligosymptomatic long-term carriers of SARS-CoV-2 display impaired innate resistance but increased high-affinity anti-spike antibodies

Elena Montes-Cobos, Victoria C. Bastos, Clarice Monteiro, João C.R. de Freitas, Heiny D.P. Fernandes, Clarice S. Constancio, Danielle A.S. Rodrigues, Andreza M.D.S. Gama, Vinicius M. Vidal, Leticia S. Alves, Laura Zalcberg-Renault, Guilherme S. de Lira, Victor A. Ota, Carolina Caloba, Luciana Conde, Isabela C. Leitão, Amilcar Tanuri, Orlando D.C. Ferreira, Renata M. Pereira, André M. Vale, Terezinha M. Castiñeiras, Dominique Kaiserlian, Juliana Echevarria-Lima, and Marcelo T. Bozza

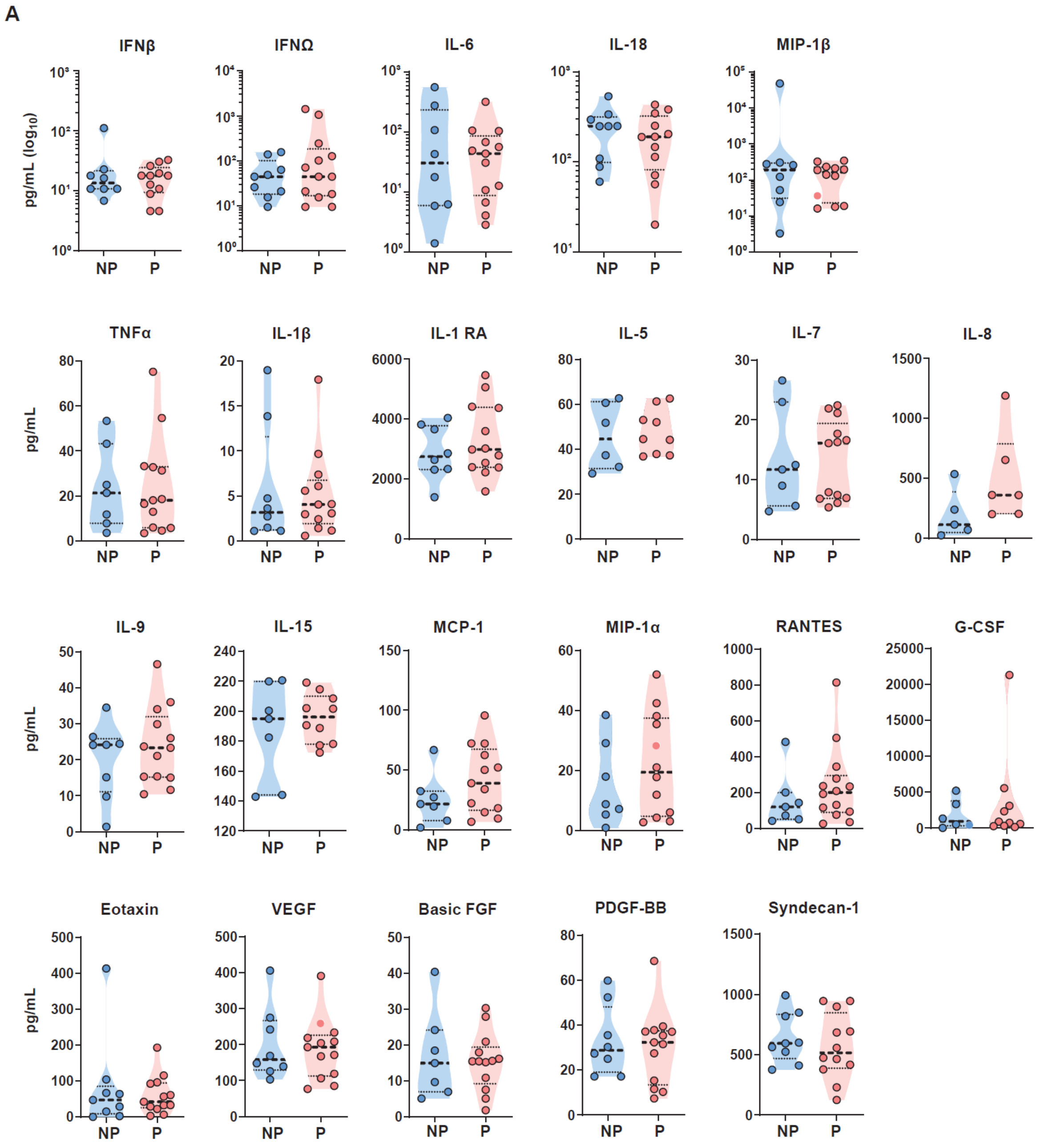

Figure S1: Cytokine analysis in swab samples at disease onset. Related to Figure 1. Cytokine quantification in swabs samples from upper respiratory tract of NP (*n*=9) and P (*n*=13) at <10 DSSO by multiplex magnetics bead-based immunoassay. Each dot represents a subject. Filled dots represent a positive qRT-PCR of nasopharyngeal samples for SARS-CoV-2. Statistical significance was calculated using Mann-Whitney test.

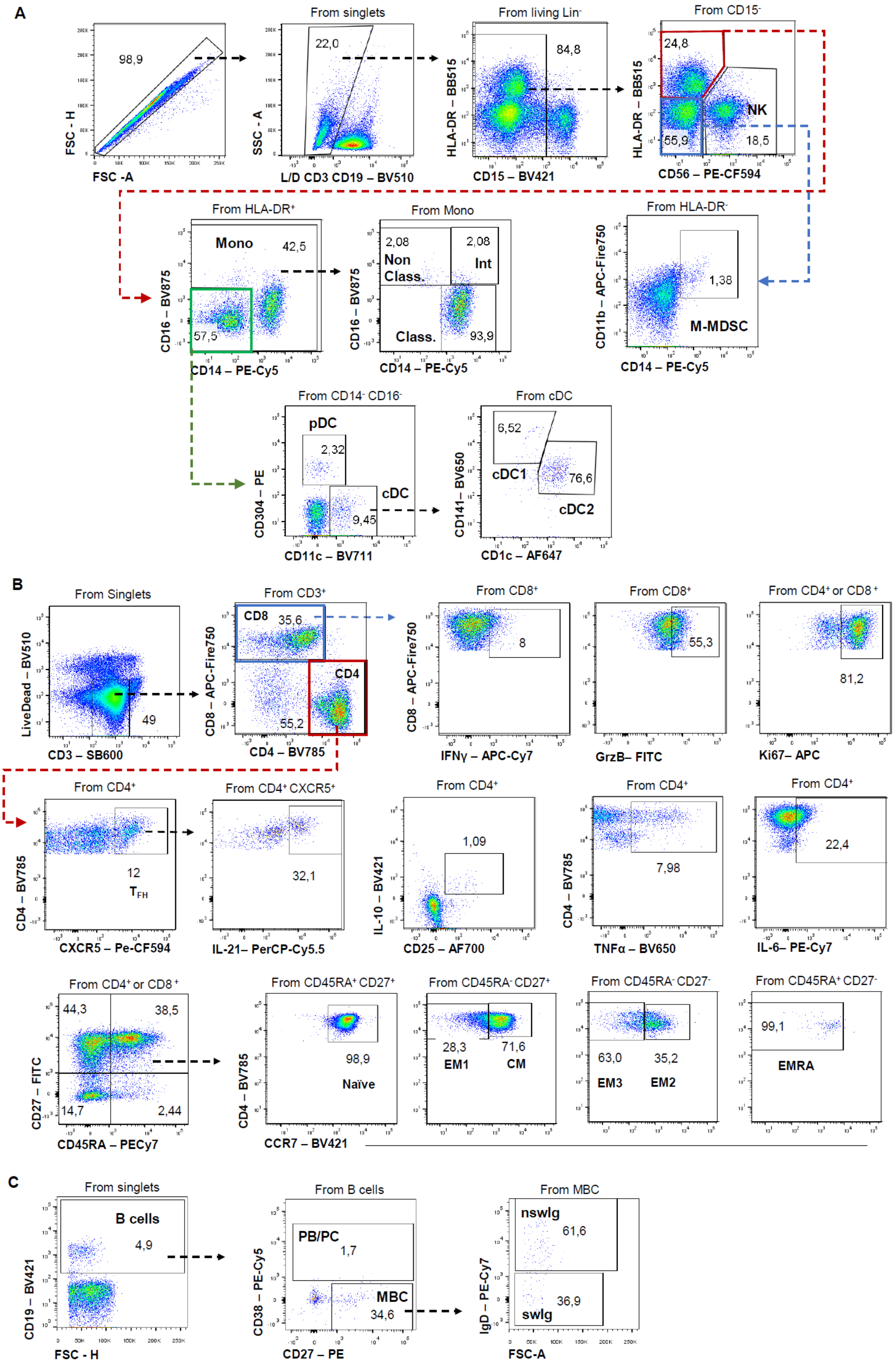
A
98,9
FSC - H
FSC -A
From singlets
22,0
SSC - A
L/D CD3 CD19 – BV510
From living Lin-
84,8
HLA-DR – BB515
CD15 – BV421
From CD15-
24,8
NK
55,9
18,5
HLA-DR – BB515
CD56 – PE-CF594
From HLA-DR+
Mono
42,5
57,5
CD16 – BV875
CD14 – PE-Cy5
From Mono
2,08
2,08
Non Class.
Int
Class.
93,9
CD16 – BV875
CD14 – PE-Cy5
From HLA-DR-
1,38
M-MDSC
CD11b – APC-Fire750
CD14 – PE-Cy5
From CD14- CD16-
pDC
2,32
cDC
9,45
CD304 – PE
CD11c – BV711
From cDC
6,52
cDC1
76,6
cDC2
CD141– BV650
CD1c – AF647
B
From Singlets
49
LiveDead – BV510
CD3 – SB600
From CD3+
CD8
35,6
CD4
55,2
CD8 – APC-Fire750
CD4 – BV785
From CD8+
8
CD8 – APC-Fire750
IFNγ – APC-Cy7
From CD8+
55,3
GrzB– FITC
From CD4+ or CD8+
81,2
Ki67– APC
From CD4+
12
TFH
CD4 – BV785
CXCR5 – Pe-CF594
From CD4+ CXCR5+
32,1
IL-21– PerCP-Cy5.5
From CD4+
1,09
IL-10 – BV421
CD25 – AF700
From CD4+
7,98
CD4 – BV785
TNFα – BV650
From CD4+
22,4
IL-6– PE-Cy7
From CD4+ or CD8+
44,3
38,5
14,7
2,44
CD27 – FITC
CD45RA – PECy7
From CD45RA+ CD27+
98,9
Naïve
From CD45RA- CD27+
28,3
71,6
EM1
CM
From CD45RA- CD27-
63,0
35,2
EM3
EM2
From CD45RA+ CD27-
99,1
EMRA
CD4 – BV785
CCR7 – BV421
C
From singlets
B cells
4,9
CD19 – BV421
FSC - H
From B cells
PB/PC
1,7
MBC
34,6
CD38 – PE-Cy5
CD27 – PE
From MBC
nswIg
61,6
36,9
swIg
IgD – PE-Cy7
FSC-A

Figure S2: Flow cytometry gating strategies. Related to Figures 2, 3, 5, and STAR Methods. Gating strategies on PBMCs for immunophenotyping of (A) innate immune cells, among them NK cells, monocytes and dendritic cells subpopulations; (B) T lymphocytes subpopulations and (C) B lymphocytes subpopulations.

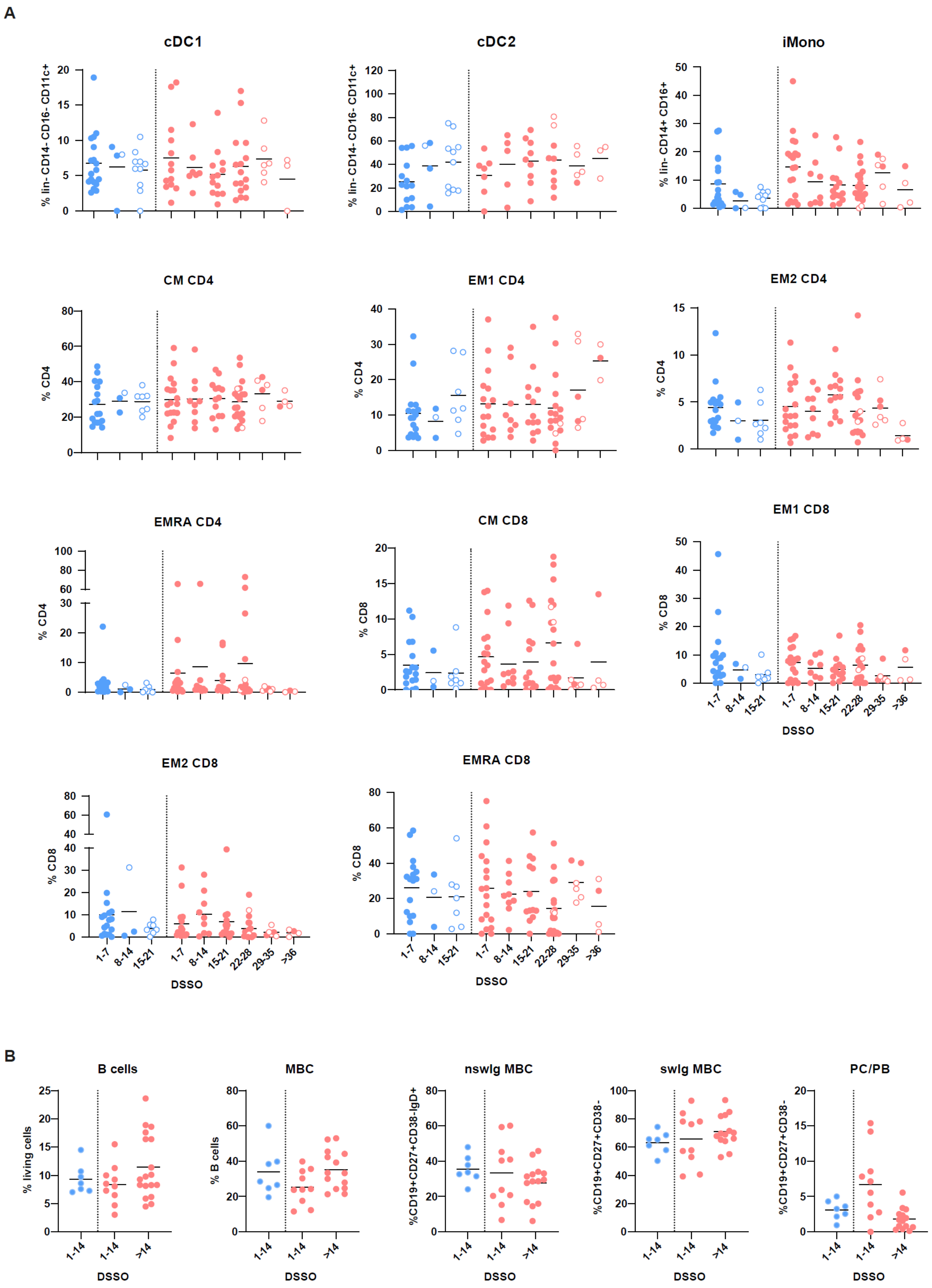

Figure S3: Longitudinal analysis of immune cell populations. Related to Figures 2, 3 and 5. Longitudinal immunophenotyping in PBMC of NP and P patients. Filled dots

represent individual samples longitudinally collected at different time points until resolution of infection from patients positive by qRT-PCR for SARS-CoV-2, while empty dots represent samples from convalescent patients coinciding with the first negative qRT-PCR for SARS-CoV-2. Statistical significance was calculated using Mann-Whitney test or Kruskal-Wallis analysis followed by Dunn post-test and indicated by *$p \leq 0.05$; ** $p \leq 0.01$; and *** $p \leq 0.001$.

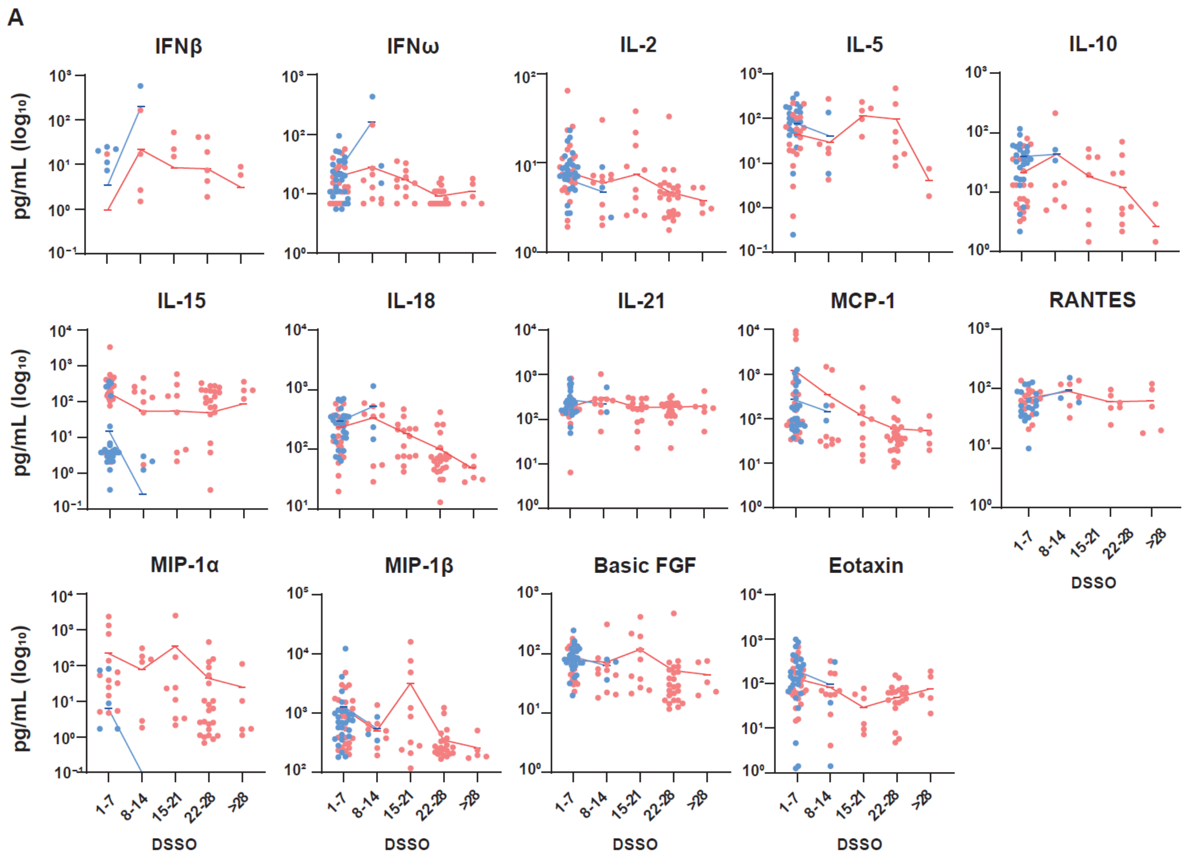

Figure S4: Longitudinal cytokine analysis. Related to Figure 2. (A) Weekly longitudinal quantification of plasma cytokines in non-persistent (NP, blue) and persistent (P, red) patients by multiplex magnetics bead-based immunoassay.

A

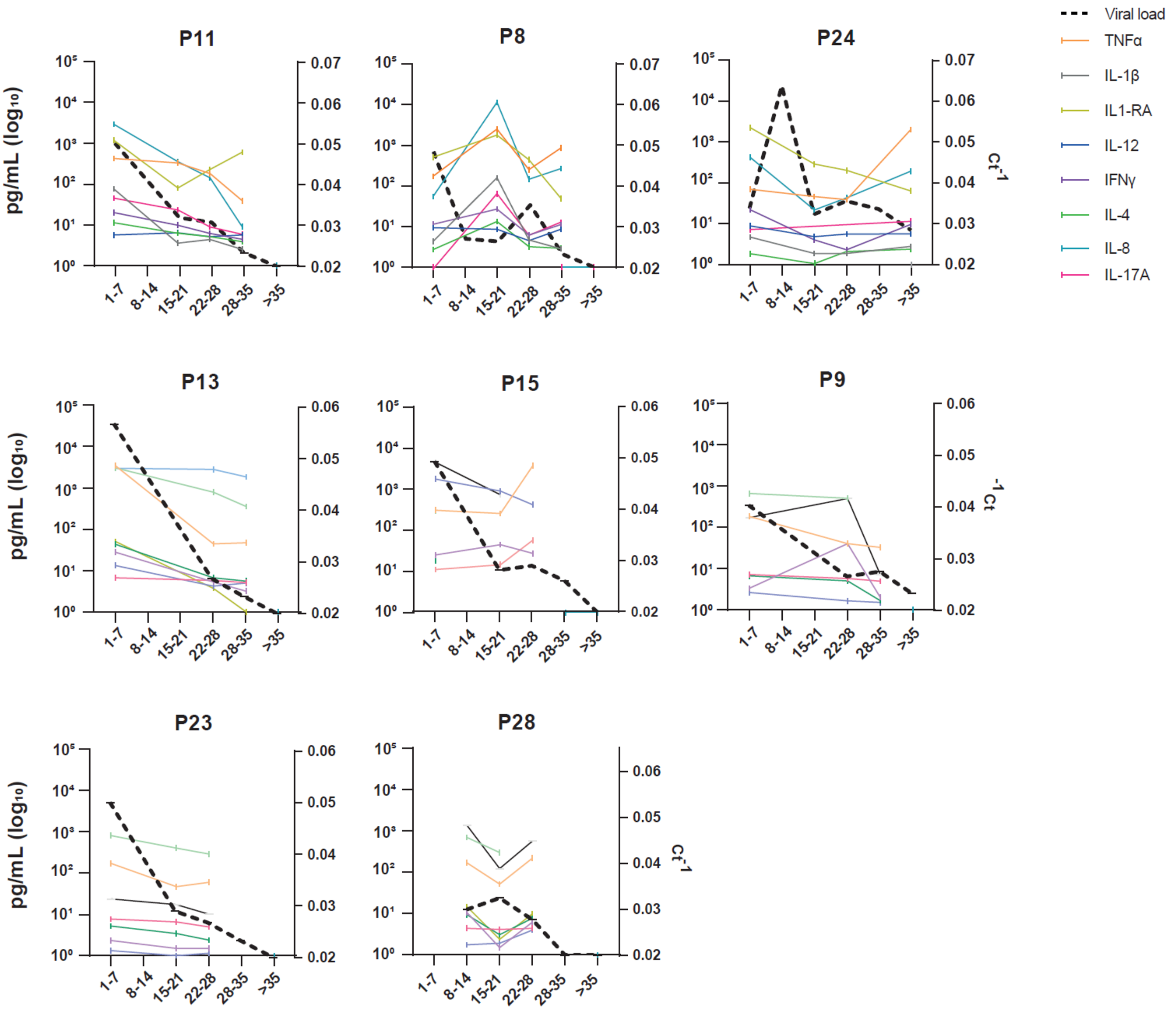

Figure S5: Longitudinal analysis of plasma cytokine and viral loads in individual patients. Related to Figures 1 and 2. (A) Longitudinal depiction of plasma IFNα, IP-10, TNFα, IL-1β, IL-12, IFNγ, IL-4, IL-9, IL-17A and IL-8 and viral loads in individual patients with persistent infection and at least three longitudinal samples available. Ct values (depicted as Ct^{-1}) were determined by qRT-PCR of viral RNA on nasopharyngeal swab samples. Cytokine concentrations were measured by multiplex magnetics bead-based immunoassay.

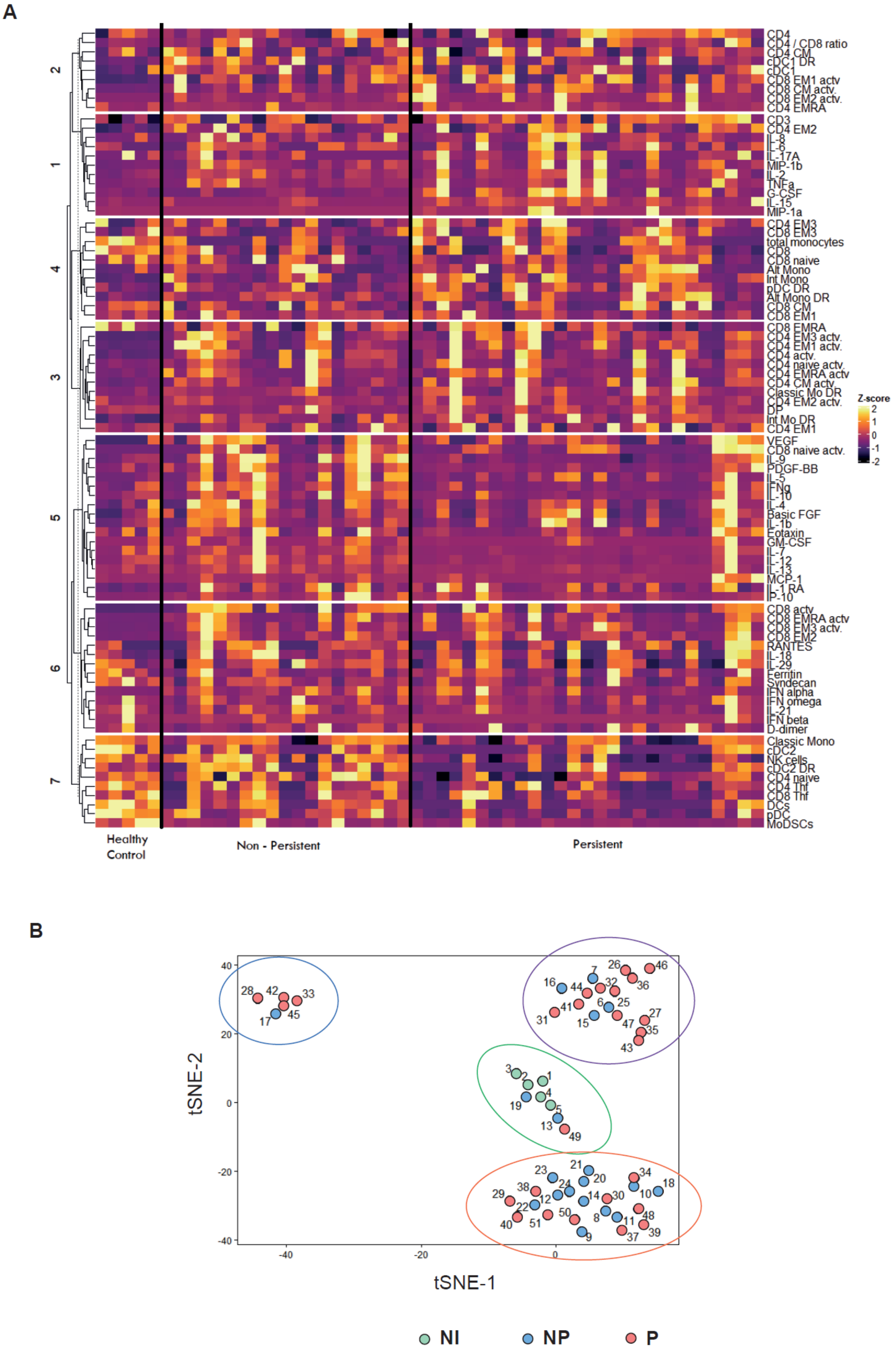

Figure S6: Cluster analysis of immunophenotyping and plasma cytokine data at disease onset. Related to Figure 4. (A) Heat map of blood cell populations and cytokine

concentration in serum from P, NP and NI at <10 DSSO measured by flow cytometry and Luminex assay. K-means clustering was used to determine cytokine clusters 1-7). Measurements were normalized across all patients by Z-score. (B) t-Distributed Stochastic Neighbor Embedding (t-SNE) probabilistic dimensionality reduction technique applied in PBMC immunophenotyping and cytokines concentration at <10 DSSO from 51 patients (P, Blue, *n*=27; NP, Orange, *n*=19; NI, Green, *n*=5). Measurements were normalized across all patients by Z-score.

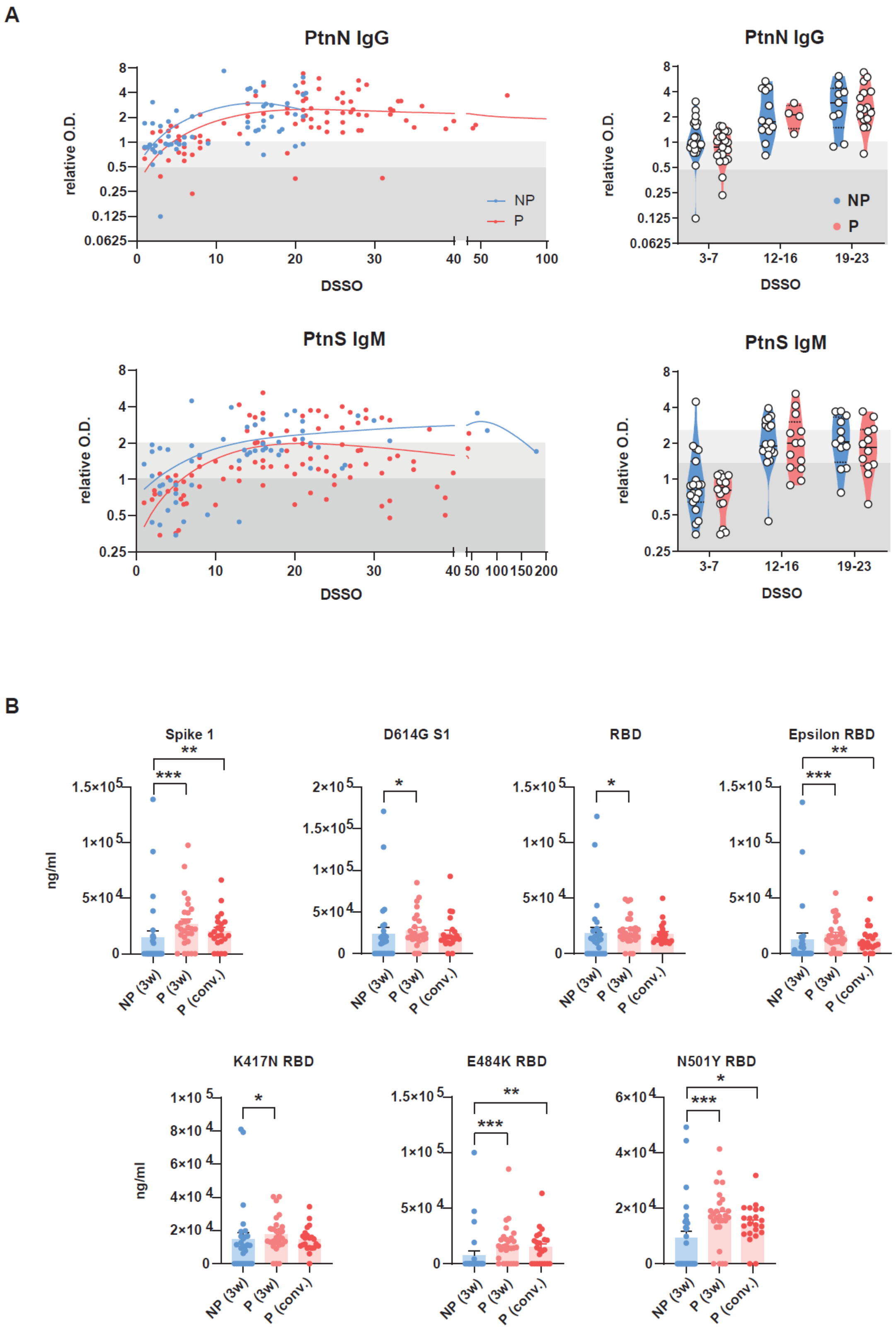
A
PtnN IgG
relative O.D.
DSSO
NP
P
3-7
12-16
19-23
PtnS IgM
B
Spike 1
D614G S1
RBD
Epsilon RBD
K417N RBD
E484K RBD
N501Y RBD
ng/ml
NP (3w)
P (3w)
P (conv.)

Figure S7: Longitudinal analysis of SARS-CoV-2-specific immunoglobulins and high affinity anti-S/anti-RBD antibodies. Related to Figure 5. (A) Longitudinal assessment of anti-N IgG and anti-S IgM antibodies in plasma from P and NP by ELISA assay. (B) Titers of high affinity antibodies against S1, D614G S1, RBD, Epsilon RBD, K417N RBD, E484K RBD, and N501Y RBD variants of SARS-CoV-2 in plasma from NP and P around 21 DSSO and P by the time of viral clearance by competitive Luminex multiplex neutralization assay. Statistical significance was calculated using Mann-Whitney test or Kruskal-Wallis analysis followed by Dunn post-test and indicated by * $p \leq 0.05$; ** $p \leq 0.01$; and *** $p \leq 0.001$. DSSO, Days since symptom onset.
