# Supplementary material for: Oligosymptomatic long-term carriers of SARS-CoV-2 display impaired innate resistance but increased high-affinity anti-spike antibodies
Source: iScience. 2023 Jun 28;26(7):107219. doi: 10.1016/j.isci.2023.107219 (PMC10300054; doi:10.1016/j.isci.2023.107219)
Supplement: Data S1. Original code used in this study, related to Figure 4 [file mmc2.zip › Covid_persistent.html]

Covid Paper Analysis


# Covid Paper Analysis

### Bioinformatic analysis of the submitted paper

#### Laboratório de Inflamação e Imunidade - UFRJ

#### 202-08-16

## Introduction

In this project, we are intending to evaluate the correlation among
pattern of cytokines expression, along with its importance in
foreshadowing wether the patient will present persistent covid or
not.

## Materials and Methods

To inspect this and maybe answer our question, our group collected
and purified the blood of several patients, isolating peripheral blood
mononuclear cells (PBMC) and cytokines. This plasma containing the
cytokines was analyzed via Luminex and the content of each of these
molecules were measured, which is the database I’m going to use in this
project.

## Loading the data

```
Planilha <-  read.csv("/Users/jramalh2/Downloads/Cytokines_complete .csv", sep = ',')
head(Planilha)
```

```
##   Sample.ID   LABINI DSSO PCR Sex Basic.FGF  Eotaxin    IFNg    G.CSF   IL.1b
## 1     HC 01      893   NA       M    18.052  62.3000  2.9120  59.9550  2.3250
## 2     HC 02      895   NA       M    18.833 142.5965 17.4140 171.2075  1.9090
## 3     HC 03 882/1717   NA       M    18.052  38.5615  3.0755 255.7480  2.1205
## 4     HC 04 879/2635   NA       M    22.747  38.9485  5.5715  11.7350  1.7030
## 5     HC 05     1818   NA       M    70.000 507.5300 84.9950 309.0000 16.8900
## 6     HC 06      913   NA       M    47.000 221.7900 57.2100 528.0000 11.6400
##    IL.1.RA   IL.2   IL.4  IL.5     IL.6   IL.7    IL.8      IL.9   IL.10  IL.12
## 1 242.0275 2.4905  1.712     0 121.0864  5.535   5.048  408.8950      NA  5.225
## 2 527.4945 2.9765  2.882     0 172.7864 10.403   5.359  348.2870  3.5560  3.622
## 3  274.345 2.2500  1.588     0 152.8650  5.669  80.241  393.3900  3.5560  3.627
## 4 260.6385 3.8980  1.212 2.401 160.5943  9.229   4.110  336.1210      NA  6.325
## 5 1,063.52 8.8300 10.090  62.3       NA  41.69 368.040 1272.7290 38.7645 29.944
## 6   354.76 5.9100  5.770 42.08       NA  41.96  14.370  997.4815 19.5150 11.618
##   IL.13   IL.15   IL.17A       IL.18       IL.21         MIF    IP.10    MCP.1
## 1 0.894   0.000  0.00000 407.7041907 284.0222686       > 900  452.406  35.3830
## 2 1.485   0.000  0.00000 279.7902366 223.3533388       > 900  166.170 171.6760
## 3 1.091  88.609  0.00000 204.2879183 277.0598824       > 900  110.036   6.8000
## 4 1.223 280.891  4.01300 99.40643223 174.1626684       > 900  102.224  33.5900
## 5 4.542   4.540  0.00000 347.8519687 181.2986439       > 900 9631.677 266.0665
## 6 2.126   2.130 10.45873 70.21258731 132.1229317 177070.9107  426.175  80.6580
##   MIP.1a   MIP.1b     TNFa   RANTES  PDGF.BB      VEGF GM.CSF IFN.alpha
## 1  1.244 222.8130  41.0950 27.09148  676.508  802.8650   0.00 2.5833645
## 2  1.100 187.2420  25.5000 41.60310 1149.982        NA   0.00 2.0946933
## 3 18.766 233.9330  32.3340 62.34858 1445.160        NA   0.00 1.8276111
## 4  0.723 191.8200  34.1910 31.70484 2548.024  547.2150   0.00 2.0946933
## 5  0.000 496.4245 247.4055 44.76176 2898.566 1312.7075   0.00 1.8276111
## 6  0.000 378.2440 140.9265       NA 3507.836  633.1665   5.04 0.6938548
##   IFN.beta IFN.omega    IL.29 Syndecan     D.dimer    Ferritin
## 1  0.00000 17.210793 841.0204 1702.846 736230.0672 1725495.063
## 2  0.00000 12.350124 865.4873 3822.621  126216.475 976068.0237
## 3  0.00000 10.381030 668.1960 2801.460 177390.5317 1406528.067
## 4  0.00000 13.041929 371.5375 1294.536 94318.67546 115570.3073
## 5  0.00000  8.181259 708.3467 4574.417  211937.411  3204297.42
## 6 35.29716  6.847878 459.9748 1392.183 18968.10338 145,076.569
```

## Organizing the data - Cytokines

```
Planilha <- Planilha[1:79,]

# Adding the patient name as rownames

rownames(Planilha) <- Planilha$Sample.ID
Planilha$Sample.ID <- NULL
Planilha$LABINI <- NULL
Planilha$DSSO <- NULL
Planilha$PCR <- NULL
Planilha$Sex <- NULL


for(i in 1:ncol(Planilha)){
  for(j in 1:nrow(Planilha)){
    Planilha[j, i] <- gsub(pattern = ',', '', Planilha[j, i])
  }
}

matrix_plani <- as.matrix(Planilha)
index <- c(which(is.na(as.numeric(matrix_plani))))
```

```
## Warning in which(is.na(as.numeric(matrix_plani))): NAs introduced by coercion
```

```
for(i in 1:length(index)){
  matrix_plani[index[i]] <- sub(pattern = ".", "", matrix_plani[index[i]], fixed = T)
}

index_2 <- c(which(is.na(as.numeric(matrix_plani))))
```

```
## Warning in which(is.na(as.numeric(matrix_plani))): NAs introduced by coercion
```

```
for(i in 1:length(index_2)){
  matrix_plani[index_2[i]] <- 0
}

citocinas <- colnames(matrix_plani)
pacientes <- rownames(matrix_plani)

numeric_matrix <- matrix(as.numeric(matrix_plani), nrow = 79, ncol = 37)

colnames(numeric_matrix) <- citocinas
rownames(numeric_matrix) <- pacientes
```

## Cytokines Heatmap

```
library(ComplexHeatmap)
```

```
## Loading required package: grid
```

```
## ========================================
## ComplexHeatmap version 2.14.0
## Bioconductor page: http://bioconductor.org/packages/ComplexHeatmap/
## Github page: https://github.com/jokergoo/ComplexHeatmap
## Documentation: http://jokergoo.github.io/ComplexHeatmap-reference
## 
## If you use it in published research, please cite either one:
## - Gu, Z. Complex Heatmap Visualization. iMeta 2022.
## - Gu, Z. Complex heatmaps reveal patterns and correlations in multidimensional 
##     genomic data. Bioinformatics 2016.
## 
## 
## The new InteractiveComplexHeatmap package can directly export static 
## complex heatmaps into an interactive Shiny app with zero effort. Have a try!
## 
## This message can be suppressed by:
##   suppressPackageStartupMessages(library(ComplexHeatmap))
## ========================================
```

```
library(colorspace)
library(RColorBrewer)

norm_data <- scale(numeric_matrix)


my_range <- function(item) {
  if(item <= -2){
    item <- -2
  } else if( item >= 2){
    item <- 2
  } 
  return(item)
}


for(i in 1:ncol(norm_data)){
  for(j in 1:nrow(norm_data)){
    norm_data[j, i] <- my_range(norm_data[j, i])
  }
}

  
  
  
  
  
heatmap <- Heatmap(matrix = t(norm_data),
                   col = colorRampPalette(rev(brewer.pal(n = 7, name ="RdYlBu")))(100),
                   name = "Z-score", 
                   show_row_dend = TRUE,
                   show_column_dend = TRUE,
                   show_row_names = TRUE,
                   show_column_names = TRUE, 
                   cluster_columns = F, 
                   cluster_rows = T,
                   km = 6, 
                   width = 900,
                   height = 700)
```

## Organizing the data - Cytokines and Cells

```
total_data <-  read.csv("/Users/jramalh2/Downloads/Complete Immunology Table - Copia.csv", sep = ',') 
 
 total_data <- total_data[1:51, ] 
 
 
 for(i in 1:ncol(total_data)){
   for(j in 1:nrow(total_data)){
     total_data[j, i] <- gsub(pattern = ' %', "", total_data[j, i])
   }
 }
 
 for(i in 1:ncol(total_data)){
   for(j in 1:nrow(total_data)){
     total_data[j, i] <- gsub(pattern = 'n/a', "0", total_data[j, i])
   }
 }
 
 for(i in 1:ncol(total_data)){
   for(j in 1:nrow(total_data)){
     total_data[j, i] <- gsub(pattern = ',', ".", total_data[j, i])
   }
 }
 

 


 pacientes <- total_data$Sample.ID
 total_data$Sample.ID <- NULL
 total_data$Sex <-  NULL
 features <- colnames(total_data)

 
 matrix_total <- as.matrix(total_data)
 
 
 dim(matrix_total)
```

```
## [1] 51 86
```

```
 data_num <- as.numeric(matrix_total)
```

```
## Warning: NAs introduced by coercion
```

```
 for(i in 1:length(data_num)){
   if(is.na(data_num[i])){
     data_num[i] <- 0
   }
 }
 
 which(is.na(data_num))
```

```
## integer(0)
```

```
 matrix_total_num <- matrix(data_num, nrow = 51, ncol = 86)
 colnames(matrix_total_num) <- features
 
 final_data <- scale(matrix_total_num)
 
 rownames(final_data) <- pacientes
```

## tSNE

```
library(Rtsne)
 library(ggrepel)
```

```
## Loading required package: ggplot2
```

```
 set.seed(10)
 
 Rtsne <- Rtsne(final_data, perplexity=5, check_duplicates = FALSE)
 rtsne_df <- as.data.frame(Rtsne$Y)
 rtsne_df$names <- rownames(final_data)
 
 rownames(rtsne_df) <- rtsne_df$names
 rtsne_df$group <- sapply(strsplit(as.character(row.names(rtsne_df)), "_"), "[[", 1) 
 
 rtsne_plot_covid <- ggplot(rtsne_df, aes(x=V1, y=V2)) + 
   geom_point(aes(fill=group), colour = "black", shape = 21, size = 4, stroke = 1) + 
   scale_fill_brewer(palette = "Set2") + 
   geom_text_repel(aes(label=rownames(rtsne_df)), size=4) +
   theme_minimal()
```

## Clustering the tSNE

```
library(clustree)
```

```
## Loading required package: ggraph
```

```
hc.norm = hclust(dist(Rtsne$Y))
rtsne_df$hclust_1 = factor(cutree(hc.norm, 1))
rtsne_df$hclust_2 = factor(cutree(hc.norm, 2))
rtsne_df$hclust_3 = factor(cutree(hc.norm, 3))
rtsne_df$hclust_4 = factor(cutree(hc.norm, 4))
rtsne_df$hclust_5 = factor(cutree(hc.norm, 5))
rtsne_df$hclust_6 = factor(cutree(hc.norm, 6))
rtsne_df$hclust_7 = factor(cutree(hc.norm, 7))
rtsne_df$hclust_8 = factor(cutree(hc.norm, 8))
rtsne_df$hclust_9 = factor(cutree(hc.norm, 9))
rtsne_df$hclust_10 = factor(cutree(hc.norm, 10))
rtsne_df$hclust_11 = factor(cutree(hc.norm, 11))
rtsne_df$hclust_12 = factor(cutree(hc.norm, 12))
rtsne_df$hclust_13 = factor(cutree(hc.norm, 13))
rtsne_df$hclust_14 = factor(cutree(hc.norm, 14))
rtsne_df$hclust_15 = factor(cutree(hc.norm, 15))
rtsne_df$hclust_16 = factor(cutree(hc.norm, 16))
rtsne_df$hclust_17 = factor(cutree(hc.norm, 17))
rtsne_df$hclust_18 = factor(cutree(hc.norm, 18))
clustee <- clustree(rtsne_df, prefix = 'hclust_')

 
 
 
 rtsne_plot_covid_2 <- ggplot(rtsne_df, aes(x=V1, y=V2)) + 
   geom_point(aes(fill=hclust_4), colour = "black", shape = 21, size = 4, stroke = 1) + 
   theme_classic()
 
 
 
 barplot <- ggplot(data = rtsne_df) + 
        geom_bar(mapping = aes(x = hclust_4, fill = group), position = "fill") +
        theme_classic()
```

## Correlation Matrix

```
library("Hmisc")
library("corrplot")
library("viridis") 
 
corr.mat <- rcorr(final_data, type = "pearson")
res1 <- cor.mtest(final_data, conf.level = .99)

#Plot correlations
correplot <- corrplot(corr.mat$r, p.mat = res1$p, type = "upper", order = "hclust", tl.col = "black", tl.srt = 45,  insig = "blank", sig.level = 0.05, col=rev(magma(200)))
```

## Importance Analysis

```
library(gtools)
library(ggplot2)
library(ggpubr)


cytokines <- read_excel("./data/raw/Cytokines complete.xls")

names <- cytokines$`Sample ID`
cytokines$`Sample ID` <- NULL
cytokines <- as.data.frame(cytokines)
rownames(cytokines) <- names

for(i in 1:length(cytokines)){
  cytokines[i] <- as.numeric(unlist(cytokines[i]))
}

cytokines_0 <- cytokines

for (i in 1:length(cytokines_0)){
  for(j in 1:79){
    ifelse(is.na(cytokines_0[j, i]),
           cytokines_0[j, i] <- 0,
           cytokines_0[j, i] <- cytokines_0[j, i])
  }
}

hc_0 <- cytokines_0[1:17,]
np_0 <- cytokines_0[18:47,]
p_0 <- cytokines_0[48:79,]


m_hc_0 <- apply(hc_0, 2, median)
m_np_0 <- apply(np_0, 2, median)
m_p_0 <- apply(p_0, 2, median)

 fc_npxp <- foldchange(m_p, m_np)

 positive <- function(x){
   if(x<0){
     return(x*-1)
   } else {
     return(x)
   }
 }
 
 module_npxp <- c()
 for(i in 1:length(fc_npxp)){
   module_npxp[i] <- positive(fc_npxp[i])
 }
 
 names(module_npxp) <- names(fc_npxp)
 
 importance_orderp <- sort(log2(module_npxp), decreasing = TRUE)
 
 top10_npxp <- as.data.frame(importance_orderp[1:10])
 
 
  top10_npxp$`Cytokines` <- rownames(top10_npxp)
  top10_npxp$`Cytokines` <- factor(top10_npxp$`Cytokines`, top10_npxp$`Cytokines`)
  colnames(top10_npxp) <- c("L2FC", "Cytokines")

 

theme_set(theme_classic())
Plot_npxp <- ggplot(top10_npxp, aes(x=Cytokines, y=L2FC)) +
  geom_point(col="tomato2", size=3) +
  geom_segment(aes(x=Cytokines,
                   xend=Cytokines,
                   y=min(L2FC),
                   yend=max(L2FC)),
               linetype="dashed",
               size=0.1) +
  labs(title="Importance Analysis",
       subtitle="Log 2 Fold Change Diferential Expression;
Non-Persistent X Persistent",
       caption="source: mpg")
```
